# Prostate Cancer Index Density, the Ratio of Percentage of Biopsy-Positive Cores to Prostate Volume, and Predicted Lethal Disease in the EAU Intermediate Prognostic Risk Class: Analysis and Implications in 651 Consecutive Patients Treated with Robot-Assisted Radical Prostatectomy at a Tertiary Referral Centre

**DOI:** 10.3390/cancers18030410

**Published:** 2026-01-28

**Authors:** Antonio Benito Porcaro, Maria Angela Cerruto, Alberto Bianchi, Riccardo Giuseppe Bertolo, Francesco Artoni, Alberto Baielli, Andrea Franceschini, Francesca Montanaro, Sonia Costantino, Alessandro Veccia, Riccardo Rizzetto, Matteo Brunelli, Salvatore Siracusano, Alessandro Antonelli

**Affiliations:** 1 Department of Urology, University of Verona, Azienda Ospedaliera Universitaria Integrata, 37129 Verona, Italy; antoniobenito.porcaro@aovr.veneto.it (A.B.P.); alberto.bianchi@aovr.veneto.it (A.B.); riccardogiuseppe.bertolo@univr.it (R.G.B.); francesco.artoni@univr.it (F.A.); alberto.baielli@univr.it (A.B.); andrea.franceschini@univr.it (A.F.); francesca.montanaro@univr.it (F.M.); sonia.costantino@univr.it (S.C.); alessandro.veccia@aovr.veneto.it (A.V.); riccardo.rizzetto@aovr.veneto.it (R.R.); alessandro.antonelli@univr.it (A.A.); 2 Department of Pathology, University of Verona, Azienda Ospedaliera Universitaria Integrata, 37129 Verona, Italy; matteo.brunelli@univr.it; 3 Department of Life, Health and Environmental Sciences, University of L’Aquila, 67100 L’Aquila, Italy; salvatore.siracusano@univaq.it

**Keywords:** prostate cancer, prostate biopsies, prostate cancer grade, percentage of positive prostate biopsy cores, prostate volume, ISUP tumour grade group system, tumour upgrading, lethal prostate cancer, EAU intermediate prognostic prostate cancer risk class

## Abstract

**Background/Objectives:** The ratio of percentage of prostate cancer (PCa) biopsy-positive cores (BPC) to prostate volume as the index density factor (Id-BPC) was used to predict the risk of high tumour grades in the surgical specimens of European Association of Urology (EAU) intermediate-risk patients treated with robotic surgery. **Methods:** From January 2013 to December 2021, we evaluated 651 patients without any prior treatment for PCa. In the surgical specimen, tumour grades were classified as indolent (International Society of Urological Pathologists Classification (ISUP) 1), significant (ISUP 2/3), and lethal (ISUP 4/5). Associations with the risk of significant and lethal cancers were assessed by the multinomial logistic regression model. **Results:** Tumour grade was clinically significant (ISUP 2/3) in 522 (80.2%) cases and lethal (ISUP 4/5) in 99 (15.2%), while the results were not significant (ISUP 1) in 30 (4.6%) subjects. The association of Id-BPC was always stronger than BPC for ISUP 1 vs. 4/5 (OR = 0.284; 95% CI: 0.128–0.632; *p* = 0.002), ISUP 2/3 vs. 4/5 (OR = 0.744; 95% CI: 0.586–0.946; *p* = 0.016), and ISUP 1 vs. 2/3 (OR = 0.382; 95% CI: 0.176–0.828; *p* = 0.015), and this trend held after adjusting for clinical factors. **Conclusions:** Accordingly, Id-BPC was positively associated with lethal disease, as, when it increased or decreased, it was more or less likely, respectively, to find ISUP 4/5 in the surgical specimens of the operated subjects, who could have been stratified according to Id-BPC risk levels.

## Simple Summary

This study evaluates the relationship between the percentage of positive prostate biopsy samples (PBS) and prostate volume, defined as the density index (Di-PBS), as a predictor of high tumour grade in surgical specimens from European Association of Urology (EAU) intermediate-risk patients undergoing robotic surgery. Between 2013 and 2021, 651 patients with no previous treatment were analysed, with tumour grades classified as indolent (ISUP 1), significant (ISUP 2/3), and lethal (ISUP 4/5). Of the patients, 82.2% had ISUP 2/3 tumours, 15.2% had ISUP 4/5 tumours, and 4.6% had ISUP 1 tumours, with Id-BPC showing a stronger association than BPC alone in predicting lethal and significant tumours (even after adjustment for clinical factors), associating higher Id-BPC values with a higher probability of ISUP 4/5 tumours, suggesting its potential role in risk stratification.

## 1. Introduction

Clinically localised prostate cancer (PCa) is actually an increasing epidemic issue for the male ageing population; accordingly, European Association of Urology (EAU) and National Comprehensive Cancer Network (NCCN) agencies continuously update guidelines with the aim to address the best management options in order to improve the quality of life in patients who need appropriate counselling [1,2]. Although clinical PCa is classified into prognostic risk groups, it still needs further stratification in order to avoid overtreatment and undertreatment, which have negative effects on oncological and functional outcomes. This issue has now become more pivotal since it has been shown that a prostate-specific antigen (PSA) test reduces PCa-specific mortality as well [3,4]. Notably, the intermediate prognostic risk class is the most frequent that occurs in clinical practice [2]. Nevertheless, it is also the most heterogenous, and management options may vary from active surveillance to treatments involving radical prostatectomy (most frequently performed by robot-assisted radical prostatectomy (RARP) and eventually associated with extended pelvic lymph node dissection (ePLND), when appropriate), external beam radiotherapy (EBRT), brachytherapy (BT), or a combination of BT and EBRT (EBRT-BT), with radiation treatments often combined with androgen deprivation therapy [5]. Therefore, further stratifying parameters are required in order to reduce both overtreatment and undertreatment issues [1,2]. Although molecular biology represents the way forward, it still has limitations in daily practice. Likewise, although multiparametric resonance imaging (mpMRI) has widely entered clinical practice and nomograms, it also has limitations because it is not reproducible in multicentre studies. The percentage of biopsy-positive cores (BPC) and tumour grades at biopsy, which are coded according to the International Society of Urological Pathology (ISUP) system, still hold as the most powerful factors informing all nomograms aiming to further stratify patients [1,2,6]. Considering this prospect, we wanted to test the hypothesis that the ratio of BPC to prostate volume (PV), which we define as the index density of BPC (Id-BPC), could be associated with the risk of adverse tumour grade (tumour upgrading at ISUP 4/5) in the radical prostatectomy specimens of EAU intermediate-risk patients who were treated with the robotic approach.

## 2. Materials and Methods

### 2.1. Features of the Investigated Population

Before 25 September 2024, we planned a retrospective observational study design, which was granted an exemption from formal approval by our Internal Review Board (IRB), in order to analyse a cohort of 651 EAU high-risk patients treated with RARP from January 2013 to December 2021, without any prior treatment, including androgen deprivation. Dedicated informed consent for data collection within the database and their use in scientific research was obtained from all patients involved in the study. Then, patients were investigated and evaluated by their age (years), body mass index (kg/m^2^), prostate-specific antigen (PSA; ng/mL), prostate volume (PV; m/L), which was measured by transrectal ultrasound, percentage of biopsy-positive cores (BPC; %), tumour grade and stage at clinical presentation, and surgical specimen. Likewise, they were assigned to the intermediate prognostic risk class according to EAU recommendations and treated with robotic surgery and eventually associated with ePLND, if appropriate. Finally, all evaluations were addressed according to guideline recommendations as well [1,2]. The risk of pelvic lymph node invasion (PLNI) was evaluated by Briganti’s 2012 updated nomogram, which applied to patients both with and without mpMRI [7].

### 2.2. Study Assumptions, Design, and Statistical Methods

We wanted to test the hypothesis that the levels of cancer density at prostate biopsies could be associated with features of adverse tumour grade in the surgical specimens of patients in the EAU intermediate prognostic risk class. Accordingly, we assumed that the ratio of percentage of BPC to PV, which we defined as the index density of BPC (Id-BPC = BPC/PV; %/mL), could be associated with lethal PCa, which was defined as tumour upgrading to ISUP 4/5. Moreover, we assumed that cancer density was a stronger predictor of tumour upgrading than standard BPC. Likewise, lethal cancer was supposed to associate with other adverse tumour features including extracapsular extension (ECE), seminal vesicle invasion (SVI), positive surgical margins (R1 stage), and pelvic lymph node invasion (PLNI). Accordingly, tumour grades occurring in the surgical specimen were categorised into three risk levels, with the first level coded as indolent or not clinically significant PCa (ISUP 1), the second level as significant cancer (ISUP 2/3), and, finally, the third level as lethal disease for tumours upgraded from biopsy ISUP 1/3 to pathology ISUP 4/5. The formulated hypotheses and assumptions were tested by statistical methods, where continuous and categorical variables were measured as a median (interquartile ranges; IQR) and by frequency (percentage; %), respectively. Differences among pathology ISUP groups were evaluated by the Kruskal–Wallis and Chi-squared statics as appropriate, while associations with the risk of lethal and significant disease were assessed by the multinomial logistic regression model (univariate and multivariate analysis). Analysis was performed using IBM-SPSS software version 26, with tests being two-sided and statistical significance indicated by *p* < 0.05.

## 3. Results

### 3.1. The Issues of Lethal and Insignificant Cancer in the EAU Intermediate Prognostic Risk Group

**Figure 1:**
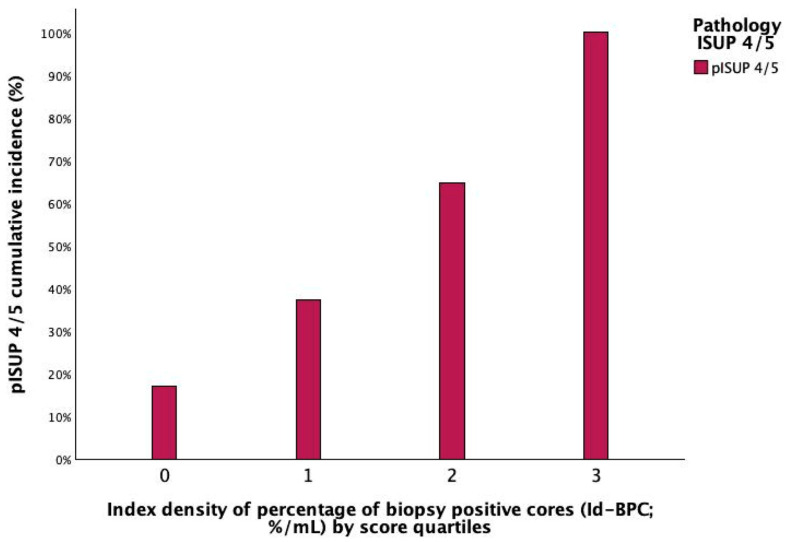
The histogram shows the positive association of tumour upgrading (pathology ISUP 4/5) predicted by the index density of percentage of biopsy-positive cores (Id-BPC), the ratio of BPC to prostate volume (%/mL), in 651 EAU intermediate-risk patients treated with robotic surgery; accordingly, as the latter increased from score 0 (up to the first quartile) to 1 (above the first quartile and up to the median) to 2 (above the median and up to the third quartile) and to 3 (above the third quartile), the rates of lethal prostate cancer increased as well. See Table 1 for further details.

Demographics of the EAU intermediate-risk population stratified by tumour grade at surgical pathology are reported in Table 1. As can be seen, tumour grade was clinically significant (ISUP 2/3) in 522 (80.2%) cases, lethal (ISUP 4/5) in 99 (15.2%), and not significant (ISUP 1) in 30 (4.6%). Tumour upgrading (ISUP 4/5) was positively associated with unfavourable clinical factors, including BPC, Id-BPC, and ISUP 3, as well as with adverse surgical pathology features, in terms of extra-prostatic extension (ECE, SVI), positive surgical margins, and PLNI as well. Figure 1 shows the positive association between tumour upgrading and Id-BPC, which has been stratified in quartile score groups. As can be seen, as the latter increased, the former was increasingly detected; conversely, the inverse association also held.

Continuous variables are reported as medians (interquartile range) and categorical factors as frequency (percentage); see also material and methods.

### 3.2. Stronger Association of Id-BPC Than BPC with the Risk of Significant and Lethal PCa

Associations of clinical and pathological factors with specimen-detected tumour grade groups are shown in Table 2. According to the clinical parameters, patients with both indolent and significant cancer, when compared with those with lethal disease, were less likely to present with higher BPC and Id-BPC values or with higher rates of ISUP grade group 3 cancers. Moreover, the former differed from the second comparison only for BPC or Id-BPC, which were both significantly lower. Nevertheless, the association of Id-BPC was always stronger than BPC for ISUP 1 vs. 4/5 (OR = 0.284; 95% CI: 0.128–0.632; *p* = 0.002), ISUP 2/3 vs. 4/5 (OR = 0.744; 95% CI: 0.586–0.946; *p* = 0.016), and ISUP 1 vs. 2/3 (OR = 0.382; 95% CI: 0.176–0.828; *p* = 0.015). Likewise, indolent and significant cancers were less likely to associate with adverse pathology features in terms of cancer extension (ECE, SVI, R1) and risk of PLNI as well.

### 3.3. Id-BPC Was an Independent Predictor of the Risk of Significant and Lethal Prostate Cancer

Table 3 shows the independent impact of Id-BPC and biopsy ISUP 3 for predicting the risk of lethal PCa after adjusting for age, BMI, PSA, and cT in the investigated population. Accordingly, patients with indolent PCa, when compared with those with lethal disease, were less likely to present with ISUP 3 (OR = 0.168; 95% CI: 0.062–0.461; *p* < 0.001) or with Id-BPC above the median and up to the third quartile (OR = 0.168; 95% CI: 0.045–0.622; *p* = 0.008) and above the third quartile (OR = 0.077; 95% CI: 0.015–0.393; *p* = 0.002) compared with cases up to first quartile. Likewise, subjects with significant disease, when compared with those with lethal PCa, were less likely to present with both ISUP 3 (OR = 0.228; 95% CI: 0.144–0.362; *p* < 0.001) and Id-BPC above the third quartile (OR = 0.499; 95% CI: 0.256–0.973; *p* = 0.041) compared with cases up to the first quartile. Nevertheless, patients presenting with indolent cancer and compared with cases presenting with significant disease were still less likely to have an Id-BPC above the median and up to the third quartile (OR = 0.270; 95% CI: 0.084–0.863; *p* = 0.027) or above the third quartile (OR = 0.153; 95% CI: 0.033–0.709; *p* = 0.016) compared with subjects up to the first quartile.

**Figure 2:**
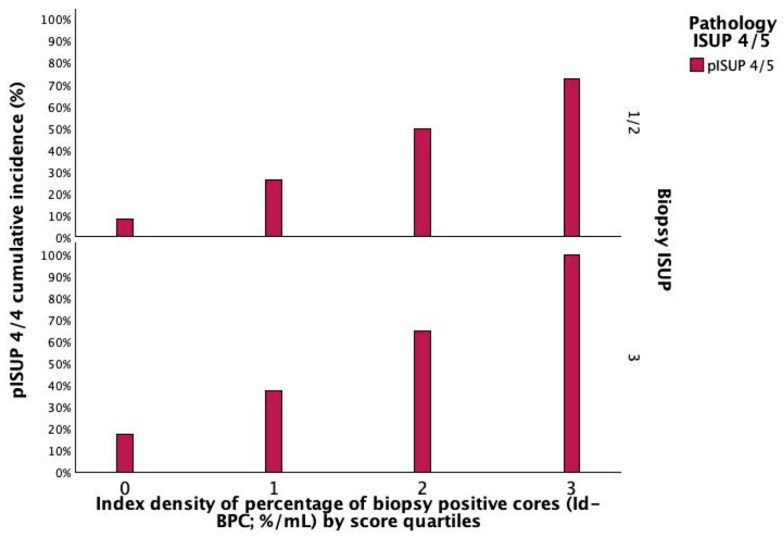
The histogram illustrates the impact of the Id-BPC quartile scores described in Figure 1 on increasing rates of lethal prostate cancer (ISUP upgraded to 4/5 in the surgical specimen); accordingly, as Id-BPC increased, patients were more likely to be found to have lethal disease in their surgical specimen independently, by presenting with or without biopsy ISUP grade group 3 as well. See the text for further details.

**Figure 3:**
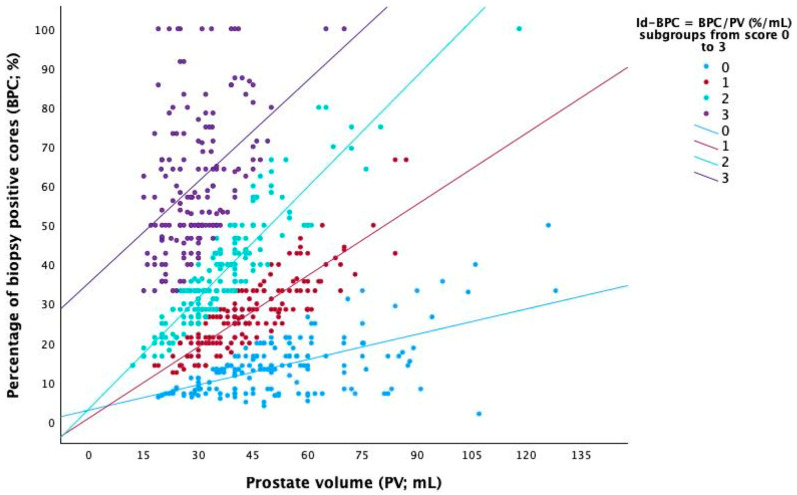
This picture depicts relationships between prostate volume (PV) and percentage of biopsy-positive cores (BPC) which predicted the index density of BPC (Id-BPC), the ratio of BPC to PV, stratified by quartiles with scores ranging from 0 to 3, as explained in the legend of Figure 1. Accordingly, as cancer densities increased from 0 to 3 (slopes of the regression lines), patients presenting with the same volumes showed increasing cancer densities, which are associated with the risk of lethal prostate cancer, as depicted in Figure 1 and Figure 2. Conversely, subjects presenting with the same percentages of BPC could be codified by quartile scores ranging from 0 to 3, with the former and the latter being associated with the largest and smallest volumes and thus relating to the lowest and highest cancer densities predicting lethal tumour grade as well. See the text for further details.

The independent impact of Id-BPC and biopsy ISUP 3 on stratifying the investigated population is depicted in Figure 2. Accordingly, as Id-BPC increased, patients were more likely to be found to have lethal disease in their surgical specimen independently, by presenting with biopsy ISUP grade group 3. Figure 3 illustrates the relationships of both PV and BPC as predictors of Id-BPC stratified by quartile scores. Accordingly, as the slopes of the regression lines increase from 0 to 3, patients are more likely to have lethal tumour grades, as explained by the legend.

## 4. Discussion

Patients classified as having intermediate-risk prostate cancer at diagnosis represent a clinically heterogeneous population that requires further stratification to identify those harbouring occult aggressive disease. Although many of these patients undergo radical prostatectomy, a subset will experience adverse outcomes, including biochemical recurrence, metastatic progression, and, ultimately, prostate cancer-specific death. Reported rates of metastatic progression range from 5% to 10%, while disease-specific mortality occurs in approximately 2% to 3% of cases. These adverse events are predicted by well-established prognostic factors, such as a short PSA doubling time and a high Gleason score (ISUP grade 4/5) at final pathology. Nevertheless, prostate cancer-specific mortality may be reduced through PSA screening, as recently demonstrated [3,8,9,10,11].

While the intermediate-risk category has been further subdivided into favourable and unfavourable subgroups, several limitations remain, including inconsistencies between EAU and NCCN classification systems, the inability of biopsy ISUP grade 2 to reliably exclude higher-grade disease, and the limited evaluation of biopsy-positive cores (BPC). Although BPC are included in all validated nomograms, they have not yet been assessed as a density parameter reflecting tumour volume, which has been shown to be a pivotal factor for risk stratification in intermediate-risk prostate cancer [1,2,12,13,14,15,16,17].

In recent years, there has been more focus on tools to improve individualised risk assessment and support shared decision-making. A recent review highlighted the growing number of risk calculators for prostate cancer [18]. These calculators can reduce unnecessary biopsies while still detecting most cases of clinically significant disease. However, there is a lack of evidence to support their routine use in all situations. Anger et al. also stressed that, despite their value, their use remains limited due to integration and standardisation issues. Most calculators rely on variables such as PSA levels and biopsy Gleason grade while ignoring quantitative parameters [19]. This can lead to different patients with similar predicted risks having very different tumour biology and clinical trajectories. There is therefore a need for new, easily measurable parameters to refine existing prediction tools, particularly for the heterogeneous intermediate-risk prostate cancer population.

Although mpMRI and quantitative imaging-derived biomarkers are promising tools for current and future clinical practice, their routine adoption remains limited by issues of reproducibility and standardisation across centres. In this context, Id-BPC represents a simple and reproducible clinical parameter that may complement imaging findings. Indeed, patients in the EAU intermediate-risk group were effectively stratified by Id-BPC quartiles, which were positively associated with lethal disease independently of biopsy ISUP grade. This approach may therefore refine risk assessment in patients both with and without detectable mpMRI lesions, including those presenting with PI-RADS scores ranging from 3 to 5.

Imaging-based artificial intelligence models are emerging as promising tools for predicting clinically significant prostate cancer prior to biopsy. A recent study demonstrated that machine learning algorithms applied to T2-weighted mpMRI acquisitions can accurately predict ISUP tumour grades. In this scenario, Id-BPC may further integrate these predictive frameworks, allowing additional stratification of both clinically significant and non-significant cancers within the EAU intermediate-risk category. Nevertheless, prospective and externally validated studies are required to confirm the clinical utility of such integrated approaches [20].

Actually, a pivotal topic is dealing with risk calculators which aim to apply an individual approach within prostate cancer pathways. Extensive reviews of the subject have shown that, while they mitigate limitations associated with early detection, the lack of external validation is a critical issue which needs to be solved by integrating them into routine clinical overflow as well. When a diagnosis of PCa is carried out, we have not only a set of parameters included into systems of risk calculators, but also others which originate from biopsy outcomes; accordingly, Id-BPC could be an effective biopsy outcome parameter to be included in future risk calculators in order to reduce the heterogeneity which impacts on management decisions in the EAU intermediate prognostic risk class. However, the inclusion of Id-BPC into new protocols defining risk calculators needs external validation in order to produce effective information when counselling this set of patients as well.

Our study has shown that, in the EAU intermediate-risk population treated with robotic surgery eventually associated with PLND, tumour upgrading was detected in 15.2% of patients, while clinically insignificant cancer was present in almost 5% of cases. Both represent a serious issue when counselling patients at the final pathology report, with the former indicating unrecognised potentially lethal cancer and the latter overtreated disease with drawbacks for the quality of life of patients. Id-BPC showed a stronger association than BPC for predicting the risk of lethal disease (ISUP 4/5) when compared with both indolent (ISUP 1) and significant PCa (ISUP 2/3), with the former also differing from the other two for higher rates of biopsy ISUP 3. This pattern was also confirmed for indolent and significant cancers, with the former differing from the latter only for the amounts of biopsy-positive cancers. So far, Id-BPC has been able to predict the risk of lethal PCa (tumour upgrading of biopsy ISUP up to 4/5), which is associated with all adverse pathology features including ECE, SVI, positive surgical margins, and PLNI. Furthermore, it allowed stratification within this prognostic risk category for predicting differences between indolent- and significant-grade tumours when compared with lethal disease as well as differences between indolent and significant PCa. As a result, these findings represent a novelty with implications for tumour biology and clinical practice as well.

Our study wanted to test the hypothesis that cancer density at biopsy could be a stronger predictor of adverse pathology than the standard percentage of biopsy-positive cores in the EAU intermediate prognostic risk class; accordingly, we assumed that more aggressive cancer biology may associate with higher cancer densities. As an example, patients presenting with the same grading (ISUP 2 or 3) and 10% biopsy-positive cores at diagnosis are intuitively not homogenous when we relate BPC to prostate volumes, which may vary from 30 mL to 60 mL or up to 90 m/L, with the former having a density of 0.33, which is higher than the 0.16 and 0.11 detected in the other two groups, respectively. Nevertheless, this parameter was compared with standard BPC, which was significantly weaker than Id-BPC for predicting lethal disease, defined as tumour upgrading (ISUP 4/5 at pathology), thus confirming the study assumptions. Moreover, prostate volume by itself did not associate with the risk of adverse tumour grade pathology as well. Finally, combining these findings, we conclude that more aggressive tumour biology is to be expected for cancers developing in smaller prostates when compared with those developing in larger prostates with lesser cancer densities as well.

Although, the concept of cancer density is intuitive, it has not yet been applied to PCa biology. Theoretically, patients presenting with 10% of BPC but volumes varying from 30 mL to 90 mL are not homogenous according to density features; indeed, the former has a higher density than the latter. As a result, it is intuitively reasonable that the former has adverse biology features developing in a microenvironment which is worse than the latter for cancers developing and progressing in larger glands as well. Interestingly, these findings are supported by a study showing that prostate volume was an independent predictor of biochemical recurrence in patients belonging to the intermediate prognostic risk class; accordingly, as prostate volumes decreased, patients were more likely to experience biochemical recurrence. However, this result did not apply to the other two risk categories. These results were explained by assuming that larger prostates have higher PSA levels, which may trigger biopsies and disease diagnosis at earlier stages, that testosterone levels dispersed in larger prostates translate to lesser aggressive activity, and, finally, that larger prostates represent a barrier to cancer extra-prostatic extension. However, the study was impaired by several limitations: it did not apply to other risk categories, it evaluated volumes in the surgical specimen but not clinically, and it did not evaluate BPC. Likewise, considering our results, we assumed that cancers developing in low prostate volumes might express a more aggressive biology for the microenvironment, which may be more hostile than those of larger ones, in terms of density hormonal levels of both testosterone and estradiol, whose dynamics we do not yet know, as well as the activity of the local immune system, which may deliver types of chronic inflammation that might or might not be protective. Accordingly, our investigation, when compared with the referred study, showed stronger results correlating clinical findings with specimen findings as well [17].

In the EAU intermediate prognostic risk class, the primary selected endpoint was tumour upgrading to ISUP 4/5, which defines lethal disease, as it represents the strongest prognostic factor for disease progression, as previously reported and discussed. In this context, Id-BPC showed a stronger association with the risk of lethal PCa than standard BPC. Although disease progression and metastasis-free survival were not included among the endpoints of the present study, Id-BPC may reasonably predict these outcomes as well; therefore, they could be considered primary endpoints in a subsequent second phase of the investigation.

Furthermore, these assumptions and findings have confirmed those of a recent preceding investigation, which we applied to the EAU high-risk category; they showed that Id-BPC, when compared with standard BPC, is a stronger independent predictor of PLNI. Accordingly, as Id-BPC increased, patients were more likely to have PLNI; conversely, as Ik-BPC decreased, subjects were less likely to be at risk of PLNI, which is the worst pathology outcome in this prognostic risk class as well [21].

So far, all issues involving the PCa intermediate risk class have had clinical implications when counselling patients; although the favourable prognostic subgroup may be managed by active surveillance, it still has the risk of adverse disease, which needs to be assessed in order to avoid delayed active treatments. Likewise, the unfavourable prognostic subgroup may hide lethal disease, which needs to be suspected in order to plan appropriate treatments related to high-risk disease. Nevertheless, when surgery is the chosen treatment, an extended PLND needs to be evaluated for those subjects who have a risk of PLNI greater than 5%. As a result, the EAU intermediate prognostic category needs sharp assessment in order to avoid risks related to under- as well as overtreatment [1,2,22,23].

Our findings support integrated clinical applications in the EAU intermediate-risk population. Patients may be initially stratified by biopsy ISUP grade and subsequently refined according to Id-BPC quartiles, which identify subgroups with progressively increasing risks of lethal tumour grades in their surgical specimens. Patients in the first two Id-BPC quartiles show the lowest likelihood of adverse pathology and may be considered for conservative management strategies. Conversely, subjects in the higher Id-BPC quartiles are more likely to harbour lethal disease associated with adverse features such as ECE, SVI, and PLNI, thus requiring extended active treatments. Moreover, Id-BPC may be incorporated into future nomograms and artificial intelligence-based predictive models in combination with advanced imaging data.

Likewise, our results show that Id-BPC was stronger than standard BPC for predicting the risk of ISUP 4/5 in the surgical specimen. As it increased, patients were more likely to have lethal disease; conversely, as it decreased, subjects were less likely to harbour occult high-grade cancer. So far, our results have implications for daily practice; patients belonging to this risk category have a 5% probability of having indolent cancer, which is predicted by biopsy ISUP 1/2 and Id-BPC low-level scores. Likewise, subjects presenting with ISUP 1/2 cancers could be stratified according to quartile level scores of Id-BPC, with risk groups for having lethal cancer varying from low to high. However, subjects presenting with grading group ISUP 3 may have Id-BPC levels in which the risk of tumour upgrading may vary from low to high as well. As a result, Figure 3 is an effective diagram for evaluating the risk of lethal disease by quartile score levels; as an example, BPCs of 10% may fall into one of the quartile risk groups according to measured prostate volume as well.

In the EAU intermediate favourable prognostic risk class, active surveillance is recommended by both EAU and NCCN [1,2]. Disease misclassification is a pivotal risk to deal with whose rates may be further lowered and managed by our results. According to our findings, patients were increasingly less likely to have indolent disease in their surgical specimen (ISUP 1 vs. ISUP 4/5) for decreasing Id-BPC quartiles, independent from tumour grade at biopsy, as shown in Table 3. These findings may lower active surveillance misclassification rates and address more effective decision algorithms within newer nomograms and/or artificial intelligence networks as well.

Our study has limitations due to its retrospective nature, because the biopsies and measurement of prostate volumes were not all performed at our institution. Furthermore, we did not evaluate targeted cores as this information was not available for all patients. All patients had at least 12 system biopsy cores and all volumes were measured by the ultrasound transrectal approach, which is the standard of care in all urologic units. Furthermore, all specimens were assessed by our dedicated pathologist as well.

From a methodological standpoint, our study investigated Id-BPC in the EAU intermediate-risk class using at least 12 systematic biopsy cores and prostate volume measured by standard TRUS methods at the time of biopsy. This represents routine clinical practice and is reproducible across institutions adopting different biopsy protocols. Moreover, the evaluation of Id-BPC density in both systematic and targeted biopsies may represent a future step toward improving biopsy strategies and further elucidating the biological behaviour of target lesions.

Potential multicollinearity diagnostics of Id-BPC with prostate volume and BPC were avoided by evaluating them separately and thus not including them in the multivariate models specifically evaluating the investigated factor. Likewise, PSA, which did not show any association with the risk of adverse tumour grade in the surgical specimen, was included in multivariate models and had no impact on odds ratios variations either.

Although prostate volume measured by mpMRI has a higher accuracy than that measured by TRUS [24], the former method was not considered because it was not available in all cases. Accordingly, prostate volume was measured by TRUS, which is the clinical standard and evaluable by the urologists themselves when performing prostate biopsies [25]. Accordingly, the impact of the bias of TRUS heterogeneity on Id-BPC reliability was minimal in the large investigated population.

Actually, the main limitation of our study is that it is single-centre/retrospective, which limits external validity. This limitation triggers confirmatory multicentre studies whose main task is to integrate Id-BPC information with other parameters, including clinical, laboratory, and imaging findings, in order to set up effective protocols applying to the EAU intermediate prognostic risk class (which stands as the most controversial because of the unexplored heterogeneity), thus impacting management decisions when counselling patients.

In our study, low Id-BPC densities were associated with a reduced risk of lethal tumour upgrading to ISUP 4/5 in the surgical specimens of a large EAU intermediate-risk population treated with robotic surgery, independently of biopsy tumour grade. This finding has relevant clinical implications and represents a major strength of the study, as it derives from a large tertiary referral centre in which all surgical specimens were consistently evaluated by a dedicated genitourinary pathologist, ensuring high internal validity and pathological accuracy. However, this same characteristic may also represent a limitation, as the single-centre design and centralised pathological assessment may limit the generalisability of our results to settings with different surgical volumes, pathological expertise, or patient selection criteria.

We did not perform decision-curve analysis as it was not the aim of the study. The clinical benefit given by BPC in decision-curve analysis has largely been demonstrated by validated nomograms, which are widely applied in clinical practice, as well [1,2].

Subgroup analyses were not adequately powered for ISUP 4/5 outcomes because of the incidence rates of such events; unexpected lethal tumour grades occurred in about 15% of the EAU investigated population and the analysis was sufficiently supported by the multivariate multinomial logistic regression analysis, which is a robust model for supporting such assessments as well [26,27,28].

Finally, we did not explore patient satisfaction. Beyond oncological outcomes, patient-reported measures have become crucial in managing prostate cancer, particularly when selecting treatment for intermediate-risk disease. A multicentre analysis of radical prostatectomy by Guercio et al. found high satisfaction but also regret for some. This was linked to outcomes differing from pre-treatment expectations. Decision regret is also associated with functional impairment, and feeling that treatment could have been avoided or is excessive [29]. These results emphasise the need for accurate pre-treatment risk assessment and patient counselling to match treatment intensity and quality of life. Improving the prognostication of intermediate-risk prostate cancer could improve treatment decisions and reduce regret and satisfaction [30].

Our study has direct implications for routine clinical practice, as it is easily reproducible across urologic units. At the time of prostate biopsy, the acquisition of at least 12 systematic cores and the measurement of prostate volume represent standard clinical practice. Within this setting, the calculation of the Id-BPC requires only a simple ratio between biopsy-positive cores and prostate volume. This allows patients belonging to the EAU intermediate prognostic risk class to be stratified into Id-BPC quartiles, which consistently reflect an increasing risk of lethal tumour upgrading from the lowest to the highest quartile, independently of the ISUP biopsy grade.

## 5. Conclusions

In the EAU intermediate PCa prognostic risk class, Id-BPC was a strong predictor of lethal disease; accordingly, as it increased or decreased, it was more or less likely, respectively, to find ISUP 4/5 in the surgical specimens of operated subjects, who could have been stratified according to Id-BPC risk levels.

## Figures and Tables

**Table 1:** EAU intermediate prostate cancer (PCa) risk class stratified by pathology tumour grade group (pISUP) of the surgical specimens after RARP.

*p*-Value	pISUP 4/5	pISUP 2/3	pISUP 1	Population	Variables
	99 (15.2)	522 (80.2)	30 (4.6)	N = 651	No. of patients
0.070	66 (61–70)	65 (60–70)	62.5 (58–67)	65 (60–70)	Age (years)
0.125	24.9 (23.2–27.2)	25.9 (23.9–27.8)	25.2 (23.5–27.8)	25.6 (23.8–27.8)	BMI (kg/m^2^)
0.791	6.6 (5.1–9.5)	6.6 (5.0–9.1)	6.4 (4.9–10.2)	6.6 (5.0–9.3)	PSA (ng/mL)
0.251	36 (28.3–45.0)	38 (30–40)	39 (30.0–52.2)	38 (30–48)	PV (mL)
<0.001	34.7 (21.4–53.3)	30 (19.6–46.6)	21.4 (12.3–28.5)	30 (18.7–47.0)	BPC (%)
<0.001	0.98 (0.63–1.61)	0.80 (0.43–1.27)	0.47 (0.29–0.77)	0.81 (0.44–1.33)	Id-BPC (%/mL)
<0.001	37 (37.4)	380 (72.2)	24 (80)	441 (67.7)	ISUP 1/2
	62 (62.6)	142 (27.2)	6 (20)	210 (32.3)	ISUP 3
0.220	52 (852.5)	305 (58.4)	21 (70)	378 (58.1)	cT1
	47 (847.5)	217 (41.6)	9 (30)	273 (41.9)	cT2
0.584	50 (42.0–65.0)	50 (40.3–62.0)	50 (45.0–64.0)	50 (45–64)	PW (gr)
<0.001	56 (56.6)	447 (85.6)	29 (96.7)	532 (81.7%)	pT2
	18 (18.2)	38 (7.3)	1 (3.3)	57 (8.8)	pT3a
	25 (25.3)	37 (7.1)	0 (0.0)	62 (9.5)	pT3b
<0.001	57 (57.6)	411 (78.7)	21 (70)	489 (75.1)	R0
	42 (42.4)	111 (21.3)	9 (30)	162 (24.9)	R1
<0.001	85 (85.9)	508 (97.3)	30 (100.0)	623 (95.7)	pN0/x
	14 (14.1)	14 (2.7)	0 (0.0)	28 (4.3)	pN1

**Table 2:** Associations of factors with pathology ISUP grade groups (pISUP) in 651 intermediate EAU prostate cancer patients treated with RARP.

	pISUP 1 vs. pISUP 2/3		pISUP 2/3 vs. pISUP 4/5		pISUP 1 vs. pISUP 4/5	
*p*-Value	OR (95% CI)	*p*-Value	OR (95% CI)	*p*-Value	OR (95% CI)	Statistics
0.103	0.957 (0.908–1.009)	0.120	0.974 (0.942–1.007)	0.022	0.932 (0.879–0.990)	Age
0.973	1.002 (0.893–1.125)	0.066	1.069 (0.995–1.149)	0.300	1.072 (0.940–1.221)	BMI
0.436	1.041 (0.941–1.150)	0.744	0.990 (0.931–1.053)	0.605	1.030 (0.921–1.152)	PSA
0.619	1.005 (0.985–1.026)	0.097	1.012 (0.998–1.027)	0.157	1.017 (0.993–1.042)	PV
0.002	0.959 (0.933–0.984)	0.010	0.988 (0.978–0.997)	<0.001	0.947 (0.921–0.973)	BPC
0.015	0.382 (0.176–0.828)	0.016	0.744 (0.586–0.946)	0.002	0.284 (0.128–0.632)	Id-BPC
0.389	0.669 (0.268–1.671)	<0.001	0.223 (0.142–0.350)	<0.001	0.149 (0.056–0.399)	ISUP 3 vs. ISUP 1/2
0.214	0.602 (0.271–1.341)	0.277	0.787 (0.511–1.211)	0.095	0.474 (0.198–1.137)	cT2 vs. cT1
<0.001	0.168 (0.073–0.385)	0.002	0.417 (0.237–0.735)	<0.001	0.070 (0.027–0.185)	PLND vs. not PLND
0.315	1.009 (0.991–1.028)	0.983	1.000 (0.988–1.027)	0.371	1.009 (0.989–1.031)	PW
0.381	0.406 (0.054–3.060)	<0.001	0.264 (0.141–0.495)	0.034	0.107 (0.014–0.844)	pT3a vs. pT2
	not applicable	<0.001	0.185 (0.104–0.331)		not applicable	pT3b vs. pT2
0.263	1.587 (0.707–3.562)	<0.001	0.367 (0.234–0.575)	0.226	0.582 (0.242–1.318)	R1 vs. R0
	not applicable	<0.001	0.167 (0.077–0.363)		not applicable	pN1 vs. pN0/x

OR, odds ratio; CI, confidence interval; see also Table 1.

**Table 3:** Independent impact of Id-BPC for predicting the risk of significant and lethal prostate cancer in the surgical specimen.

pISUP 1 vs. pISUP 2/3	pISUP 2/3 vs. pISUP 4/5	pISUP 1 vs. pISUP 4/5	
OR (95% CI)	OR (95% CI)	OR (95% CI)	Statistics (*)
			*Id-BPC by quartiles:*
0.847 (0.360–1.993)	0.822 (0.401–1.685)	0.696 (0.239–2.027)	second vs. first quartile
0.704	0.592	0.056	*p-value*
0.270 (0.084–0.863)	0.621 (0.315–1.227)	0.168 (0.045–0.622)	third vs. first quartile
0.027	0.170	0.008	*p-value*
0.153 (0.033–0.709)	0.499 (0.256–0.973)	0.077 (0.015–0.393)	fourth vs. first quartile
0.016	0.041	0.02	*p-value*
			*ISUP at biopsy*
0.738 (0.291–1.872)	0.228 (0.144–0.362)	0.168 (0.062–0.461)	ISUP 3 vs. ISUP 1/2
0.523	<0.001	<0.001	*p-value*

OR, odds ratio; CI, confidence interval; (*) after adjusting for age, BMI, PSA, cT; Id-BPC: index density of percentage of biopsy-positive cores; ISUP: International Society of Urologic Pathology system for grading prostate cancer; see also Section 2.

## Data Availability

Data are contained within the article.

## Abbreviations

The following abbreviations are used in this manuscript:

BPC	Biopsy-Positive Cores
EAU ECE	European Association of Urology Extracapsular Extension
Id-BPC	Index Density of Biopsy-Positive Cores
ISUP	International Society of Urological Pathologists Classification
mpMRI	Multiparametric Magnetic Resonance Imaging
PCa	Prostate Cancer
PLNI	Pelvic Lymph Node Invasion
PV	Prostate Volume
RARP	Robot-Assisted Radical Prostatectomy
R1	Positive Surgical Margin
SVI	Seminal Vesicle Invasion
